# Combined Influence of Meso- and Macroporosity of Soft-Hard Templated Carbon Electrodes on the Performance of Li-O_2_ Cells with Different Configurations

**DOI:** 10.3390/nano9060810

**Published:** 2019-05-28

**Authors:** Mara Olivares-Marín, Mohamed Aklalouch, Dino Tonti

**Affiliations:** 1 Departamento de Ingeniería Mecánica, Energética y de los Materiales, Universidad de Extremadura, Centro Universitario de Mérida, 06800 Mérida, Spain; maraom@unex.es; 2 Institut de Ciència de Materials de Barcelona, Consejo Superior de Investigaciones Científicas (ICMAB-CSIC), Campus UAB, ES 08193 Bellaterra, Spain; m.aklalouch@uca.ma; 3 Laboratory of Chemistry of Materials and Environment, Faculty of Sciences and Technology, University Cadi Ayyad, Bd. Abdelkrim El Khattabi, B.P. 618 Guéliz, 40100 Marrakesh, Morocco

**Keywords:** meso-macroporous carbons, resorcinol-formaldehyde xerogels, pore size distribution, lithium-air battery, cell configuration

## Abstract

Li-O_2_ batteries can offer large discharge capacities, but this depends on the morphology of the discharged Li_2_O_2_, which in turn is strongly affected by the nanostructured carbon used as support in the air cathode. However, the relation with the textural parameters is complex. To investigate the combined effect of channels of different sizes, meso-macroporous carbons with similar mesopore volume but different pore size distribution were prepared from the polymerization of resorcinol-formaldehyde (RF) in the presence of surfactants and micro-CaCO_3_ particles. The carbon materials were used as active materials of air cathodes flooded by ionic liquid-based electrolytes in Li-O_2_ cells with two different configurations, one with a static electrolyte and the other with a stirred electrolyte, which favor a film-like and large particle deposition, respectively. The presence of large pores enhances the discharge capacity with both mechanisms. Conversely, with respect to the reversible capacity, the trend depends on the cell configuration, with macroporosity favoring better performance with static, but poorer with stirred electrolytes. However, all mesoporous carbons demonstrated larger reversible capacity than a purely macroporous electrode made of carbon black. These results indicate that in addition to pore volume, a proper arrangement of large and small pores is important for discharge capacity, while an extended interface can enhance reversibility in Li–O_2_ battery cathodes.

## 1. Introduction

Many studies based on the impact of porous carbon-based air cathodes architecture on Li-O_2_ battery performance coincide with the fact that the discharge capacity increases with pore volume especially when pore sizes are within the mesoporous range [1,2,3]. However, the literature offers contradictory results on the optimal pore sizes for large discharge capacities. Younesi et al. [4] and Ma et al. [2] found that pore sizes larger than 30 nm could be considered useful to increase the discharge capacity. Mirzaeian at al. [1] tested a wide range of carbon aerogels and found the highest discharge capacity when the average pore size was 14.23 nm. Nimon et al. [5] and Kuboki et al. [6] noticed that the deposition of solid discharge products occurred within the pore volume rather than on their surface; however, while the former noticed the filling of pores with radii of up to 10 nm, the latter found that large open volumes such as those of Super P carbon black are suitable for Li-O_2_ batteries. Also, Tran et al. [7] reported that micropores and smaller mesopores would be blocked at the beginning of the discharge reaction, and solid Li oxides reside inside the larger pores. In effect, Ding et al. [8] reported that pore sizes of 80 nm are optimal for a large discharge capacity. Alternatively, Yang et al. [9] showed an important increase in discharge capacity when they used bimodal mesopores with a narrow pore size distribution, centered at 4.3 and 30.4 nm, compared to several carbon blacks. Accordingly, Zhang et al. [10] found an increased capacity by combining the carbons of different porosity. Such contradictions may be understood by considering the variety in the morphology of discharge products obtained in different conditions of electrolytes, cells, and applied electrochemical conditions. In effect, we have shown that the discharge mechanism and the consequent morphology of the deposits is a major factor to consider before determining an optimal electrode architecture [11]. Factors like current density [12,13,14,15,16], electrolyte [17,18,19,20], and cell design [11] can significantly determine whether a surface Li_2_O_2_ film forms or larger particles grow from the solution phase. When a 5–10 nm film forms, the ideal pore diameter is twice this thickness, for which pores between 20–40 nm are optimal [21]. Instead, when the mechanism changes to solution-phase growth, the same materials are much less effective than structures with high porous volume such as Super P carbon black, able to host large amounts of precipitate [11]. However, this general principle only takes into account the way that the space offered by the electrode can be filled by discharge products of a given morphology. In practical cases, discharge is often affected by reactant depletion, which in turn may locally switch the deposition mechanism. This can significantly complicate comparison between electrode materials of very different porous networks and stresses the relevance for all the cases of wide channels across the electrode thickness. In fact, several authors [22,23] have reported that a network throughout the electrode formed by micron-sized macropores can enhance the O_2_ transport and, therefore, result in high discharge capacity. Modeling has confirmed this concept although a proper balance between macroporosity and microporosity is required for optimal results [24]. Most of these studies only consider the impact of porosity on the discharge capacity, but not on rechargeability.

In this work, we have investigated the combined influence of Li-O_2_ cell configuration and electrode porosity range on the discharge capacity and rechargeability. We have developed a purely disordered mesoporous material and two meso-macroporous materials having large voids which connect highly mesoporous areas with uniform pore diameters. For comparative purposes, Super P carbon black was also used as an example of a merely macroporous material with a high open porous structure. These materials were used as anactive material of air cathodes in Li-O_2_ cells with two different configurations, one with a static electrolyte and the other with a stirred electrolyte. An ionic liquid (IL)-based solution (i.e., 1-methyl-1-butylpyrrolidinium bis(tri-fluoromethane sulfonyl)-imide (PYR14TFSI) with lithium bis(trifluoromethylsulfonyl)imide salt (LiTFSI) was employed as the electrolyte. These kind of electrolytes have been widely used for rechargeable Li-O_2_ batteries [25,26] and present some interesting properties such as high conductivity, non-flammability, non-volatility, and wide temperature ranges of operation [27,28,29,30,31]. Furthermore, several studies have shown that some IL-based electrolytes are more stable, compared to other organic electrolytes, to the superoxide radical anion attack [32], and that their use could lower overpotentials [25,33], while increasing the rechargeability of the Li-O_2_ cells [25,34].

## 2. Materials and Methods

### 2.1. Mesoporous Carbon Preparation

Based on other procedures earlier reported [35,36], a mesoporous carbon (MC) was prepared through the self-organization of surfactants and carbon precursors (resorcinol-formaldehyde, RF), followed by carbonization. Briefly, 0.1 mL of HCl (37%, Sigma Aldrich, Madrid, Spain) and 1.4 g of a non-nonionic surfactant Pluronic^®^ F127 (Sigma Aldrich) were firstly dissolved in 10 mL of ethanol (99%, Panreac, Barcelona, Spain) in a polypropylene container. After their complete dissolution, 1.4 g of resorcinol (≥99.0%, Sigma Aldrich, R) was added. The mixture was stirred at room temperature for 10 min. Subsequently, 2 g of formaldehyde solution (37% in H_2_O, Sigma Aldrich, F) was introduced drop by drop while stirring. In this way, the molar ratios on the solution were fixed to R:F = 1:2 and F127:R = 1:1. The mixture was stirred for about 30 min and then kept under static conditions uncovered at room temperature in a fume cupboard to evaporate ethanol (around 48 h). The residue was heated at 100 °C in the same plastic container sealed for 24 h in order to promote the polymerization between R and F. The resulting orange solid was grounded and carbonized at 900 °C for 1 h under an argon stream with a constant flow rate of around 100 mL min^−1^. The sample was then allowed to cool down to room temperature under the same argon flow. The resulting product was sieved in order to homogenize the carbon particles.

### 2.2. Meso-Macroporous Carbon Preparation

**Figure 1:**
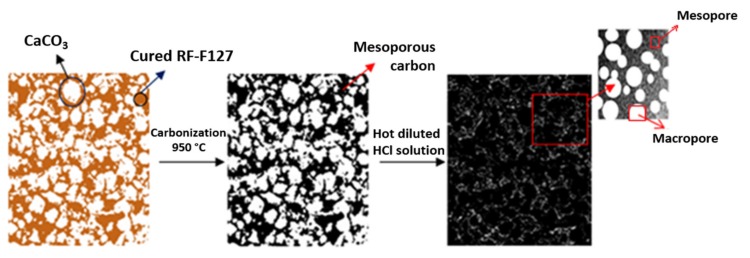
Scheme of the methodology followed for the preparation of MMC-1 and MMC-2 carbons.

**Figure 2:**
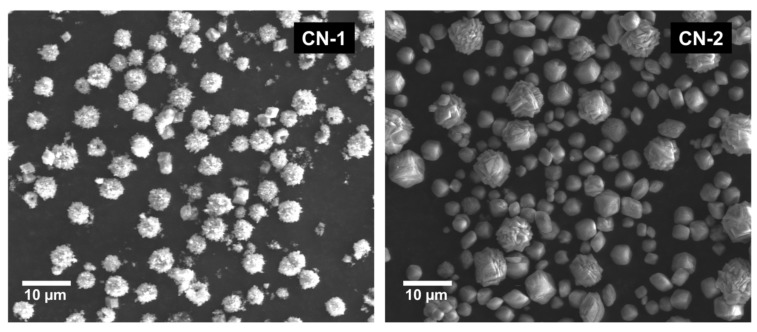
SEM images of CN-1 (**Left**) and CN-2 (**Right**) CaCO_3_-based microparticles.

Meso-macroporous carbons were prepared by a similar procedure as employed for MC carbon from a mixture of ethanol (24 mL), R (0.175 g), F (0.25 g), F127 (0.175 g), and 0.8 g of spherical microparticles of CaCO_3_ (labeled as CN-1 and CN-2, see below) as macroporous templates without adding HCl. Sample MMC-1 was prepared with CN-1 and MMC-2 with CN-2. This resulted in the approximate molar ratios R:F = 1:2, R:F127 = 1:1, and R:CN = 1:4.5. After the carbonization, carbons obtained were washed with hot 0.1 M HCl solution for removing CaCO_3_ particles and then with hot distilled water. Finally, the resultant products (MMC-1 and MMC-2) were dried at 100 °C overnight. Figure 1 illustrates the general method followed for the preparation of these carbons. CN-1 and CN-2 CaCO_3_-based microparticles were obtained using two different methods. CN-1 particles were prepared by precipitation of calcium carbonate from equimolar portions of CaCl_2_ (99.99% trace metals basis, Sigma Aldrich) and Na_2_CO_3_ (≥99.0%, Sigma Aldrich) under stirring in 200 mL in the presence of the anionic surfactant sodium dodecylbenzenesulfonate (1 g L-1, SDBS, technical grade, Sigma Aldrich) following similar procedures as reported by Hwang et al. [37]. Briefly, 100 mL of 0.1 mol of NaCO_3_ and 0.2 g of SDBS were mixed under stirring at room temperature. Once SDBS was totally dissolved, 100 mL of 0.1 mol of CaCl_2_ was added to the previous solution, and the new solution was kept under stirring for at least 1 h. Later, the precipitates were separated by centrifugation (4000 rpm for 15 min) and washed with distilled water and ethanol. The obtained CaCO_3_-based microparticles were dried at 60–80 °C for 12 h. On the other hand, CN-2 particles were synthesized through the hydrolysis of dimethyl carbonate (DMC, anhydrous, ≥99%, Sigma-Aldrich, St. Louis, MO, USA) as a source of CO_2_ in an aqueous solution according to a procedure reported by Faatz et al. [38,39]. Briefly, 400 mL of 0.001 mol of CaCl_2_ (anhydrous, 99.99% trace metals basis, Aldrich) aqueous solution, including 0.005 mol of DMC and 2.637 g of poly(sodium 4-styrenesulfonate (PSS, average Mw ~70,000, Sigma Aldrich) were mixed with 100 mL of 0.5 molars of NaOH (≥98.0%, Sigma) aqueous solution. The mixed solution was stirred for about five min at room temperature. The precipitates were separated by centrifugation (4700 rpm 15 min) and, afterward, washed using water, acetone, and ethanol. The obtained solids were dried under a vacuum condition at room temperature for 48 h. CN-1 microparticles (see Figure 2) resulted with narrow particle diameter distribution (3 μm–6 μm), while CN-2 had a wider distribution (400 nm–13 μm).

### 2.3. Porous Carbon Characterization

Texture and porosity of porous carbons were analyzed by scanning electron microscopy (SEM, FEI Quanta 200 FEG-ESEM, Hillsboro, OR, USA), transmission electron microscopy (TEM, JEOL JEM1210, Tokyo, Japan, operated at 100 keV), and N_2_ adsorption/desorption (Micromeritics ASAP 2020, Norcross, GA, USA). Details of the characterization procedure and textural data determination are provided in previous reports [21,40]. Pore size distributions (PSD) were estimated by the Barrett–Joyner–Halenda (BJH) method [41]. The predominant pore size (D_max_) was taken as the pore size corresponding to the maximum of the N_2_ adsorption PSD.

### 2.4. Electrode Preparation and Electrochemical Tests

The different porous carbons were ground in a mortar and sieved through a 180-mesh stainless steel mesh. Carbon powder (80 wt.%) was mixed with 10 wt.% of polyvinylidene fluoride (PVDF) as a binder and 10 wt.% of carbon black (Super P, Timcal, Bodio, Switzerland) in N-methylpyrrolidone (NMP, Sigma Aldrich). The slurry obtained was used to impregnate a stainless steel mesh (AISI316, 180 mesh per inch, ADVENT Research Materials Ltd., Witney, UK) and finally was dried at 100 °C for 12 h under vacuum. The electrode loading was of the order of 0.5 mg cm^−2^.

**Figure 3:**
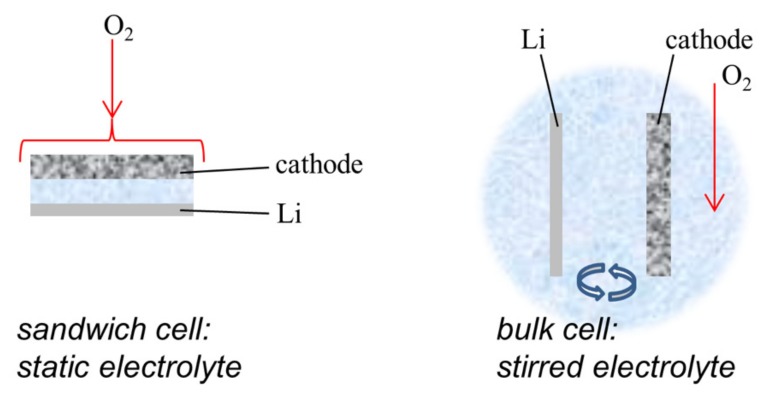
Sketch highlighting the main aspects of the two cell configurations used in this study.

The electrochemical tests were performed using two different cell configurations in which the oxygen electrode was completely wetted with the electrolyte (see Figure 3). A cell-based on ISO-KF high-vacuum components [40] will be referred to here as the “sandwich” cell. The separator used for this cell was a glass fiber filter (GFPC grade, 270 µm thick, PRAT DUMAS, Couze-et-Saint-Front, France) soaked with ~100 μL of electrolyte. The negative electrode used for this cell was a 10 mm lithium metal disk (Sigma-Aldrich, 0.4 mm thick). The other cell, described in more detail previously [11], was based on a three-necked 5 mL flask and called the “bulk” or stirred electrolyte cell. In this case, two necks were used for the anode and the cathode, respectively, and the third one was reserved for both the inlet and outlet for the bubbling of the O_2_ gas. The negative electrode (lithium metal) was encapsulated inside a capillary glass with an aperture in contact with the electrolyte to avoid the direct contact of lithium with O_2_ gas. The electrodes had a distance of around 1 cm in the electrolyte that was stirred with a magnetic bar. The electrolyte used for this study was a mixture of IL 1-methyl-1-butylpyrrolidinium bis(trifluoromethane sulfonyl)-imide (PYR14TFSI, Solvionic, Toulouse, France, purity 99.5%) with lithium bis(trifluoromethylsulfonyl)imide salt (LiTFSI, 99.95%, Sigma-Aldrich) with a PYR14TFSI:LiTFSI molar ratio of 1:9. PYR14TFSI was stored in a dry box and used as received, while LiTFSI was dried at 120 °C under vacuum for 48 h before use. The water content of the electrolyte was below 20 ppm, as checked with a Metrohm KFC 899 Coulometric Karl Fischer titrator. The cells were placed in a home-made thermostated chamber at 60 °C. Pure O_2_ flow (5 mL/min) was forced to pass continuously through the cell 30 min before starting and during the electrochemical measurements, which were operated with a Bio-Logic VMP3 multi-channel potentiostat.

## 3. Results and Discussion

### 3.1. Porous Carbon Characterization

**Figure 4:**
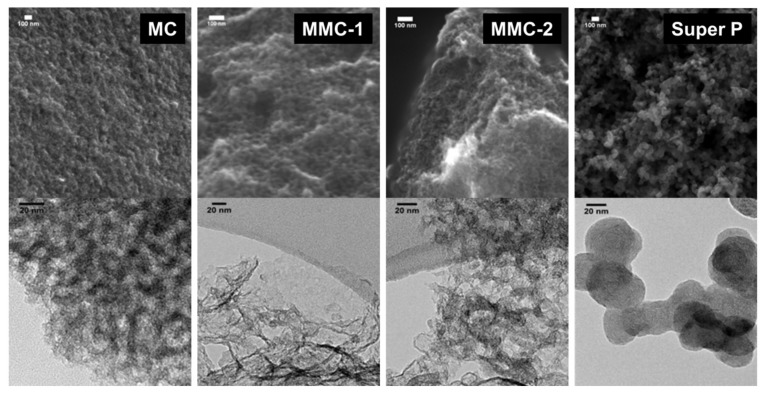
Representative SEM (**Top**) and TEM (**Bottom**) micrographs of MC, MMC-1, MMC-2 and Super P carbons.

Texture and porosity of porous carbons were analyzed by scanning and transmission electron microscopy (SEM and TEM), showing remarkably different morphologies (see Figure 4). Sample MC shows a disordered mesopore structure, but quite uniform in pore diameter. In fact, wormhole mesopores are clearly visible in the TEM image and these are about 10 nm in diameter. MMC-1 and MMC-2 possess a porous structure composed of two different kinds of pores. Some uniform mesopore structures are connected by large pores or voids with various shapes and sizes (from 0.4–20 μm), which can be attributed to the presence of the spherical CaCO_3_ templates. The macropores of MMC-1 seem more connected and giving place to a more open structure than MMC-2. Moreover, both samples clearly appeared to be less dense than MC. Instead, the Super P electrode consists of a well-opened structure with a wide distribution of pores in the macropore range up to about 500 nm. This is originated from its chain-like structure resulting from the combination of graphitic pseudo-spheroidal nanoparticles (~40 nm). 

**Figure 5:**
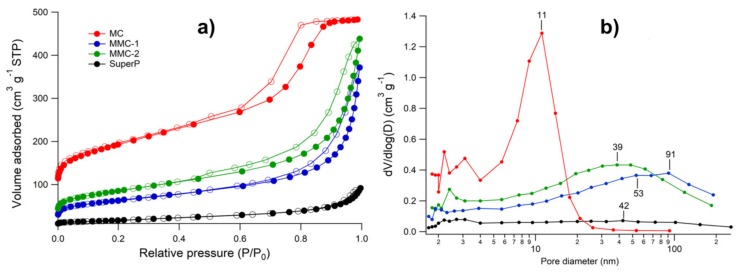
(**a**) N_2_ adsorption/desorption isotherms at −196 °C of MC, MMC-1, MMC-2 and Super P mesocarbons and (**b**) their corresponding BJH pore size distributions.

Figure 5a compares nitrogen adsorption/desorption isotherms at −196 °C for the carbon materials. Table 1 summarizes the main textural data obtained from N_2_ adsorption/desorption isotherms. MC carbon presents a Type IV isotherm exhibiting an H1 hysteresis loop, which means that this material is essentially a mesoporous carbon with channel-like mesopores [35,42]. MC possesses an apparent surface area of 685 m^2^ g^−1^ (see Table 1) that is in good agreement with typical values obtained for other analogous of mesocarbons [35]. On the other hand, MMC-1 and MMC-2 curves can be assigned to Type II isotherms, corresponding to macroporous materials. However, the hysteresis loops may indicate the presence of an important amount of mesopores. In fact, as it can be seen in Table 1, MC, MMC-1, and MMC-2 exhibit similar mesopore volume, V_meso_, of around 0.5 cm^3^ g^−1^. Nevertheless, the PSD curves (Figure 5b) of these samples indicate important differences in the predominant pore size. Thus, while MC exhibited a narrow pore size distribution centered at around 11 nm, MMC-2 and MMC-1, exhibited wide pore size distributions centered at 41 nm and 72 nm, respectively. The carbonate particles seem to significantly interact with the surfactant, and depending on their size the resulting mesopore distribution is considerably shifted towards larger sizes. The significantly higher microporous volume of MC vs. MMC-1 and MMC-2 could instead be ascribed to a higher resin polymerization pH when carbonate particles were used.

These results confirm that the CaCO_3_ hard templates have allowed the formation of large and heterogeneous pores among highly mesoporous areas. By using smaller commercial CaCO_3_ particles (30–50 nm), Li et al. [22] have reported in previous work the synthesis of micron-sized honeycomb-like carbon material. As a carbon precursor, they used sucrose, and the proportion of mesopores in the resulting carbon structure were around 78%. In our case, we have used RF and Pluronic F127 surfactant to ensure a highly mesoporous area uniform in pore diameter, interconnected by large and heterogeneous channels created by using our CaCO_3_ particles. As a result, the proportion of mesopores obtained in our case was around 95%. On the other hand, Super P is a well-known non-porous material with a moderate apparent surface area (60 m^2^ g^−1^) and very small total pore volume (~0.14 cm^3^ g^−1^, see Table 1). Also, it presents according to Figure 5b a PSD maximum of 40 nm that could be related to interstices between particles forming their chain-like structure. Therefore, our set of samples can be considered a range from a completely open structure to an increasingly extended mesoporous network in the order: super P < MMC-1 < MMC-2 < MC. 

### 3.2. Electrochemical Tests

To understand the role of the different types of pores in relevant operating conditions, the set of our carbons, we tested them in in static conditions providing a range from a disordered mesoporous material with uniform pore size (MC) to a meso-macroporous material having very large and heterogeneous channels which connect highly mesoporous regions (MMC-1).

**Figure 6:**
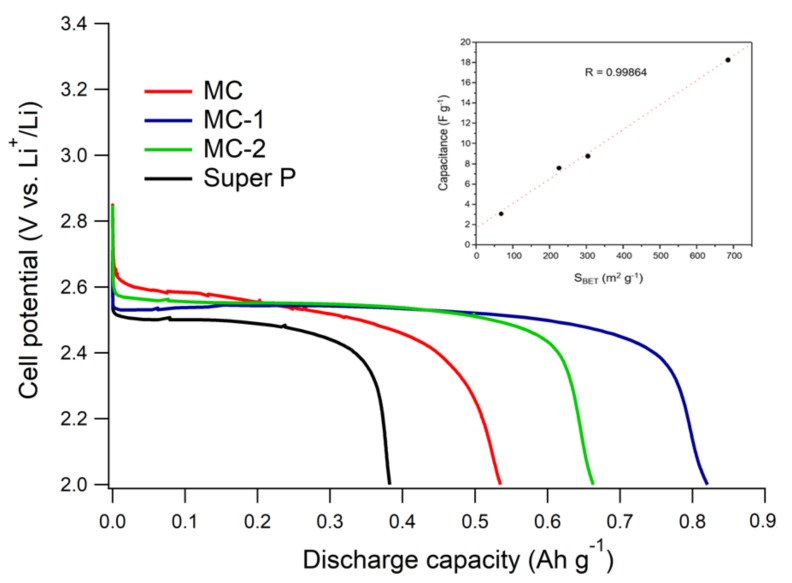
Galvanostatic measurements of carbons/SS mesh electrodes at 60 °C at 0.1 mA cm^−2^ using a sandwich cell configuration and (inset) a graph plotting the capacitance (F g^−1^) versus S_BET_ data.

Figure 6 shows the discharge curves of carbon-based electrodes at 0.1 mA cm^−2^ and 60 °C using a static IL-based electrolyte cell using the static configuration (i.e., sandwich cell type). The maximum capacity expected for the different carbons, based on their mesopore volume, is 1350 mAh g^−1^ for MCs carbons and 378 mAh g^−1^ for Super P. These values were obtained by multiplying the mesopore volume (≈0.50 cm^3^ g^−1^ for mesocarbons and 0.14 cm^3^ g^−1^ for Super P) by the theoretical capacity for dense Li_2_O_2_ (ca. 2700 mAh cm^−3^) [43]. This value does not consider the interparticle volume in the electrode but can be an estimation for the capacity that can be expected by each porous material in ideal discharge conditions. The micrometric-sized interstitial porosity should not significantly contribute with its nm-thick film-like discharge on large pores. 

From Figure 6, MCs carbons did not reach the expected capacities. Instead, Super P did. This fact confirms that the static system presents severe limitations in oxygen mass transport when using an IL-based electrolyte. Nevertheless, from the results in this specific system, the following points are noteworthy. As shown in Figure 6, the initial discharge voltage correlates well with the specific area. As it could be expected, the capacitance, calculated from the initial slope before the plateau, has an excellent linear correlation with area (inset in Figure 6). This is consistent with a purely double-layer capacity, and with earlier observations [40,44] regarding the positive effect of using highly available surface areas to drive a smaller overpotential during discharge. On the other hand, carbons should display a high surface area from pores wider than the ion size for a high capacitive response [28]. In this case, pores wider than 1.5 nm (ion diameter of PYR14-based IL [28]) predominate in the porous carbons, and therefore, the surface area exhibits a direct correlation with capacitance, which also shows that a large part of the electrode surface is electrochemically active before discharge. However, previous work has shown that high surface areas and highly developed pore volumes do not necessarily deliver larger discharge capacities [9,21,22,45]. Also, in the present case, discharge capacity was more dependent on the actual pore size distribution than on the mesopore volume or apparent surface area. This is because, in our conditions, Li_2_O_2_ mainly precipitates as a few nm-thick conformal films on the carbon surface and does not fill most pores, as previously shown [21].

**Figure 7:**
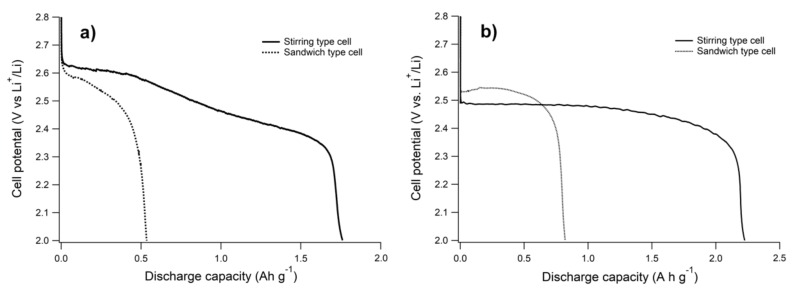
Discharge capacities of MC (**a**) and MMC-1(**b**)/SS mesh electrodes at 60 °C at 0.1 mA cm^−2^ using a sandwich and a bulk cell type.

From Figure 5b and discharge profiles in Figure 6, it can be appreciated that mesoporous samples with the average pore size localized in the macropore range provided the highest discharge capacities, which resulted in the order MC < MMC-2 < MMC-1. This order did not follow the specific area but the maximum of the pore size distribution of MC carbons, which otherwise had similar V_me_ (≈0.50 cm^3^ g^−1^). This result confirms that mesoporous carbons with very large and heterogeneous channels or macropores can enhance the contribution of smaller pores enabling an efficient O_2_ and Li^+^ transport [8,21,22,23]. However, even if wide channels are desirable, mesoporosity plays an important role in our regime. In fact, Super P, where small-sized pores are scarce, delivered a very low discharge capacity compared to MCs carbons. With a solution-mediated discharge, involving 3D deposits, the requirements change [11,25,46,47,48]. We have tested the same series of electrodes also using a bulk stirred electrolyte cell, which according to our previous studies with IL-based electrolytes, enables a switch of the discharge mechanism from nm-thick film to micrometric particles [11], obtaining considerably larger capacities, although overpotentials are generally larger due to the large iR drop of this cell (see Figure 7). Due to its high surface area, MC showed mainly a discharge curve without potential plateau, while MMC-1 showed a long plateau indicating the formation of Li_2_O_2_ with a small variation of the electrochemically active area. This suggests a better pore connectivity in the latter case, which also results in better filling of the available volume.

**Figure 8:**
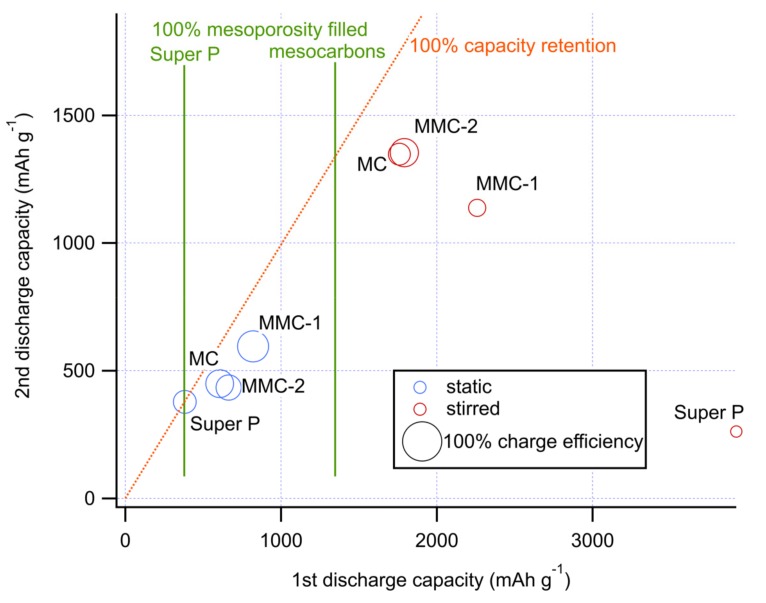
Diagram representing the capacity for the 2nd vs. 1st full discharges in the static (blue) and stirred (red) electrolyte cells. Circle diameter is proportional to charge/discharge coulombic efficiency. The dotted line represents total capacity retention between the first two discharges, green vertical lines represent capacities calculated assuming complete mesoporosity filling by Li_2_O_2_ for Super P and the mesocarbons prepared in this study.

Figure 8 summarizes the capacities obtained for all carbons in both cell configurations, also considering first charge and second discharge, which are significant indicators for reversibility and capacity retention. Discharge capacities for all materials using the stirred bulk cell exceeded those delivered with the static cell, with a three-fold and ten-fold increase for MCs carbons and Super P, respectively. The fact that the capacity delivered when the electrolyte is stirred exceeded the value based on mesopores only (1350 mAh g^−1^, based on the corresponding volume), implies deposition in macropores and interstices between carbon particles are in agreement with the expected thick irregular formation of Li_2_O_2_, rather than a thin conformational film. Also, with the bulk cell, we observed a positive effect of the extra macroporosity created with CaCO_3_ templates, with larger discharge capacities from materials with more open structures. The discharge capacity obtained for Super P using the bulk cell type was comparable to values reported by Elia et al. [25]. With its low area and large open volume, Super P performs poorly with a film-forming mechanism, but is ideal for a volume-filling deposition, raising an enormous difference in capacity compared to the mesoporous carbons. 

We have previously developed models to relate pore size distributions with discharge capacities within a series of carbons using either of the two cells [11,21], which can take into account either surface or solution mediated discharge mechanisms. We were not able to apply them with a satisfactory correlation to the present carbon series in the respective cells. Given the different carbon textures (as observed by electron microscopy Figure 4), we believe that the different contribution of macropores in the carbon particle or in the electrode itself can create different accessibility, and possibly even a locally different mixture of both discharge mechanisms. 

The reaction reversibility is also strongly affected by both cell and carbon texture. The Coulombic efficiency was generally higher in the case of the static electrolyte, as the thin Li_2_O_2_ layer can be easily decomposed during the charge due to a sufficient electron transfer from carbon. Subsequently, a smaller capacity loss is observed at the second discharge. Instead, in the stirred-electrolyte cell, the thick deposit fills most of the available volume, and therefore, the electron transfer from carbon will be more limited during charging, leaving a higher amount of low-conductivity by-products [8,11,49]. Consequently, a more severe capacity loss is expected. The trend of a larger first discharge corresponding to a smaller second discharge is however not strictly respected. For the sandwich cell system, the MMC-1 sample, having the most open structure, showed the best discharge capacity, charge coulombic efficiency (79%), and retention at the second discharge (73%). On the contrary, when using the stirred bulk cell type, this sample MMC-1 delivered poorer battery rechargeability and capacity retention, while samples with less open structure favored capacity retention (up to 77%). Considering the severe capacity loss suffered by Super P, this suggests that filling of wide channels generates too large particles that are poorly electrically connected and thus difficult to remove. As a result, all mesocarbons consistently deliver superior second discharge capacities than Super P. However, reversibility is not strictly related to retention; MC and MMC-2 in the bulk-type cell present similar discharge but different charge capacities (coulombic efficiency of 56% and 72%, respectively). In this case, the larger microporous volume of MC may be easier to clog irreversibly, but without obstructing the remaining volume, which remains accessible through the interstitial channels within the electrode. This suggests that size distribution and arrangements of the porous network can even intervene in the reversibility of electrode reactions, which are important factors on the battery cycle life. 

Overall, within our study, the best performance is obtained with a stirred electrolyte, mainly favoring a volume discharge mechanism, and a cathode architecture including both wide channels and significant mesoporosity. 

## 4. Conclusions

By combining soft and hard template routes, we prepared different porous carbon materials with similar mesopore volume but ranging from a disordered mesoporous material with uniform pore size to a meso-macroporous material having very large and heterogeneous channels which connect highly mesoporous domains. When used as air cathodes in different cell configurations, the presence of large pores or voids enhanced the first discharge capacity. We attribute this benefit to improved O_2_ transport, which further expands the electrode utilization even when the electrolyte is stirred. Remarkably, this effect is more important than the specific area. On the other hand, with respect to charge efficiency and retention, the trend depends on the configuration, in fact, the sample with a pore size maximum at 72 nm showed the best behavior with a static cell system but the worst with a stirred bulk cell. This suggests that mesoporosity, by better dispersing the deposits, favors reversibility with a volume phase deposition mechanism, when the capacity is larger.

Even if the specific electrode architecture providing optimal performance in Li–O_2_ batteries depends on the operating conditions, this study suggests that in general, compared to an open purely macroporous structure, a mesoporous carbon offers better balance between the first discharge capacity and reversibility, allowing larger second full discharge capacities, regardless of the cell used. Macro-meso and purely mesocarbons presented here provided remarkable capacity retention even after full discharge with a solution-phase mechanism, and are therefore interesting candidates for sustained cycling.

## Figures and Tables

**Table 1:** Textural data of carbons. Abbreviations: S_BET_, Brunauer–Emmett–Teller (BET) surface area; V_micro_: micropore volume; S_ext_: external surface area; D_max_: predominant pore size; Vtotal: total pore volume; Vmeso: mesopore volume; %Vme: percentage of mesopore volume.

Sample	S_BET_ (m^2^ g^−1^)	V_micro_ (cm^3^ g^−1^)	S_ext_ (m^2^ g^−1^)	D_max_ (nm)	V_total_ (cm^3^ g^−1^)	V_meso_ (cm^3^ g^−1^)	%V_meso_ (%)
MC	685	0.11	429	11	0.64	0.53	83
MMC-1	225	0.03	163	72	0.53	0.50	94
MMC-2	303	0.04	224	41	0.63	0.59	94
Super P	67	-	70	40	0.14	0.14	100
